# Supplementary figures and images for: National trends and socioeconomic inequalities in the Composite Index of Severe Anthropometric Failure among children under five in Bangladesh
Source: PLOS Glob Public Health. 2026 Feb 26;6(2):e0005881. doi: 10.1371/journal.pgph.0005881 (PMC12944787; doi:10.1371/journal.pgph.0005881)

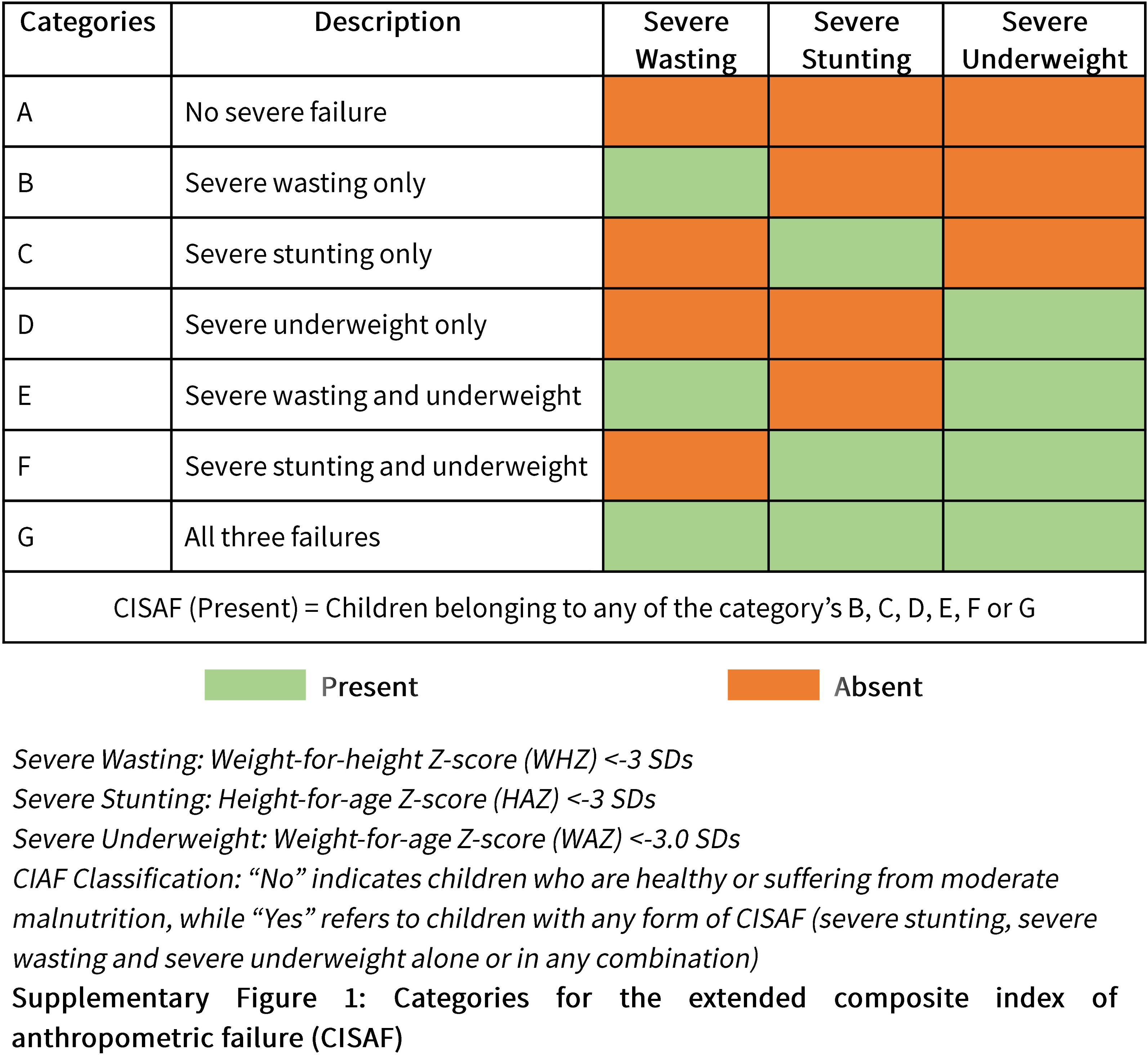

Supplement: S1 Fig — (TIF) [file pgph.0005881.s001.tif]

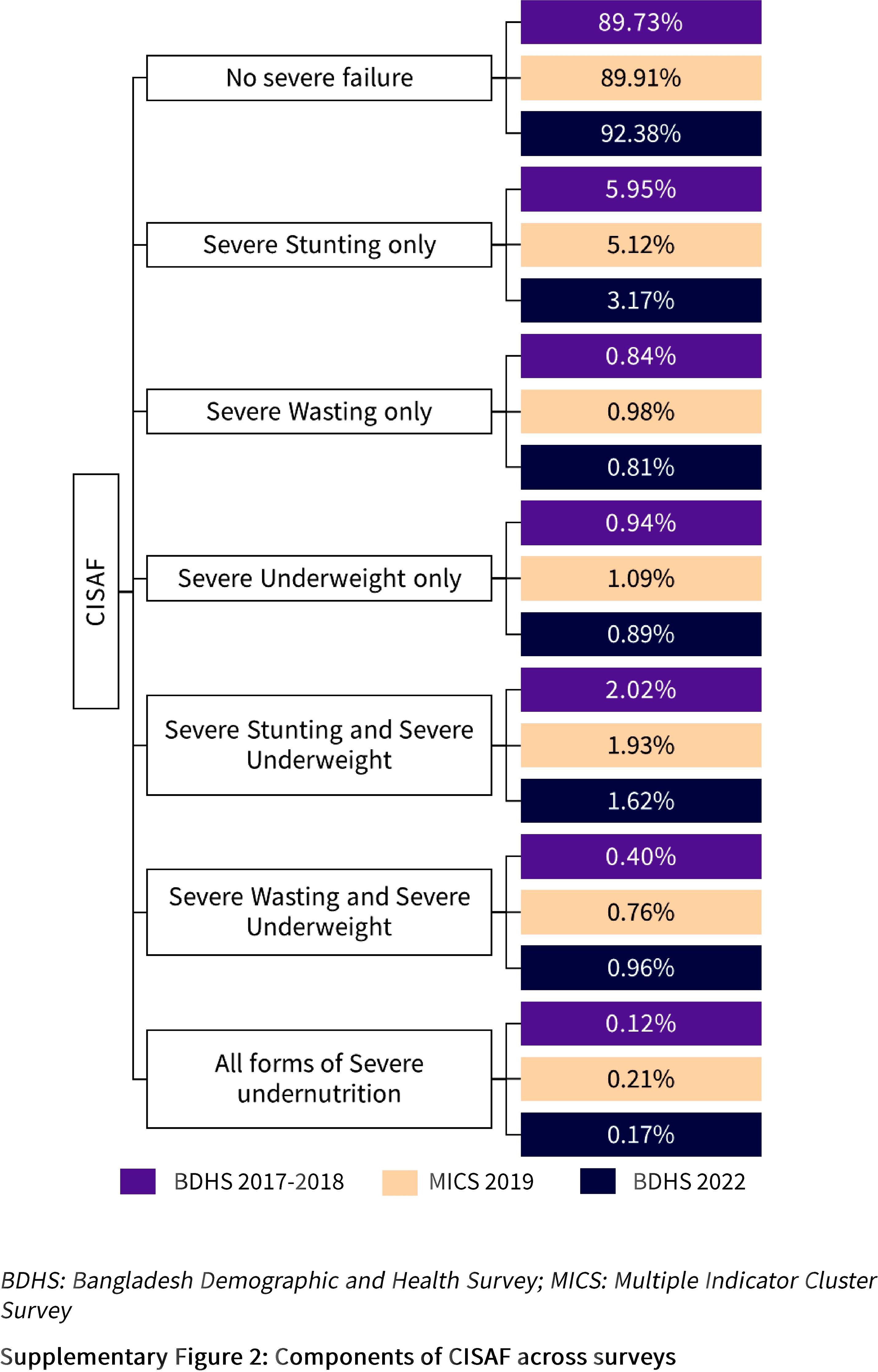

Supplement: S2 Fig — (TIF) [file pgph.0005881.s002.tif]
