# Supplementary material for: National trends and socioeconomic inequalities in the Composite Index of Severe Anthropometric Failure among children under five in Bangladesh
Source: PLOS Glob Public Health. 2026 Feb 26;6(2):e0005881. doi: 10.1371/journal.pgph.0005881 (PMC12944787; doi:10.1371/journal.pgph.0005881)
Supplement: Attachment — Submitted filename: Review Report_01.pdf [file pgph.0005881.s004.pdf]

Date: 23.11.2025

Journal Name: PLOS Global Public Health

Manuscript Title: National Trends and Inequalities in Severe Anthropometric Failures in Bangladesh: Evidence from National Surveys

Manuscript Number: PGPH-D-25-02016R1

Thank you very much for giving me the opportunity to review the manuscript "National Trends and Inequalities in Severe Anthropometric Failures in Bangladesh: Evidence from National Surveys". The relevance of this paper is notable, particularly in the context of Bangladesh. It utilises nationally representative data to explore nutritional disparities between boys and girls under the age of five. This research can increase scientific understanding. However, I recommend a thorough major revision to improve the manuscript's presentation and overall impact.

REVIEWER COMMENTS

SECTION-WISE OBSERVATION

Title: This is partially acceptable

- It lacks relevance and does not adequately reflect the study's objective. I recommend revising the title to better align with the content of the research. Additionally, while the study focuses on CISAF, the mention of "Severe Anthropometric Failures" seems misplaced and should be clarified or adjusted accordingly.

Keywords: This is partially acceptable

- Please avoid repeating terms from the title in the keywords section.
- Acronyms such as "DHS," "MICS," "SII," and "RII" should be spelled out to enhance understanding, as they may not be familiar to all readers.

Abstract: Partially Acceptable; moderate corrections are required

- You have collected data from BDHS 2017-18, MICS 2019, and BDHS 2022, leading to a trend analysis covering a relatively short time span of only five years. For a more robust analysis, I recommend using data spanning at least a decade.
- Is this study intended as a longitudinal study? You note that "Higher odds of CISAF were associated with increasing age of the child (aOR: 1.03, 1.10 and 1.02, respectively)." suggesting that children become CISAF as they grow older. This warrants further clarification or write the sentence correctly.
- Regarding your statement, "However, lower odds were linked to wealthier families and higher education of the mother." while maternal education is undoubtedly significant, it would enhance your analysis to include paternal education as well.
- I suggest clarifying and refining the conclusion for a more precise presentation of your findings.

Introduction: Acceptable, but major corrections are required

- In the sentence "To overcome this, Svedberg proposed the Composite Index of Anthropometric Failure (CIAF), which incorporates all forms of failure.[1]" the reference is cited as Anik et al. (2021). It seems more appropriate for this claim to be attributed to Svedberg. Could you please clarify this?
- In your discussion regarding CISAF, you mention "Later, Vollmer et al. (2017) introduced the...in resource-poor settings.[5]". However, the reference here is listed as Saif and Anwar (2023). Please review this and ensure that proper attribution is given.
- I recommend that you recheck all the references thoroughly, as the arrangement appears to be inconsistent. A systematic review of citations would enhance the manuscript's credibility.
- It may be beneficial to include further references to support your study. This could help substantiate your claims and provide a broader context for your findings.

Objective: Partially Acceptable; minor corrections are required

- The objective should be revised to provide a clear statement that reflects the title of the study.
- The text states, "Using three recent nationally representative surveys..." However, there is no mention of the names of these surveys. It would be helpful to include this information to provide clarity and context for the reader.

Methods: Partially Acceptable; moderate corrections are required

- The definition of implausible values for Z-scores appears to deviate from standard ranges, instead of "Implausible values were defined as weight-for-height Z-score (WHZ) <-5 or >5 SD, height-for-age Z-score (HAZ) <-5 or >5 SD, and weight-for-age Z-score (WAZ) <-6 or >5 SD.". Please refer to the WHO Multicentre Growth Reference Study Group's guidelines: "WHO child growth standards: length/height-for-age, weight-for-age, weight-for-length and body mass index for age: methods and development. Geneva: World Health Organization, 2006." Specifically, implausible values should be based on accepted thresholds for weight-for-height Z-score (WHZ), height-for-age Z-score (HAZ), and weight-for-age Z-score (WAZ).
- I noticed inconsistencies in the terminology used to refer to the target population. It would be beneficial to adopt a single term throughout the manuscript, such as "under-five children," "U5C," or "under five," to enhance clarity.
- The focus of this study is on "Inequalities"; however, the sex of the child (male/female) is utilized as an independent variable. I suggest a more thorough exploration of the potential implications of this variable in the context of inequalities.
- Additionally, I recommend including a section labelled "Study variables" that distinctly outlines the "Outcome variable" and the "Independent variable." It would also be beneficial to provide "Operational definitions for the variables" separately to enhance understanding.

Results: Partially Acceptable, major corrections are required

- I recommend conducting the analysis separately for boys and girls, as the focus is on "inequalities." This approach may reveal factors that are specifically related to each gender, providing a deeper understanding of the issues at hand.

Discussion: Acceptable; minor corrections are required

- It may be beneficial to include further references to support your study.

Conclusion: This is acceptable

References: This is acceptable

OVERALL OBSERVATION

The suggestion for improving the language to avoid the use of "we" or "our" in the manuscript is well-founded. By opting for a more formal and objective tone, the authors can enhance the clarity and professionalism of their work.
