# Supplementary material for: National trends and socioeconomic inequalities in the Composite Index of Severe Anthropometric Failure among children under five in Bangladesh
Source: PLOS Glob Public Health. 2026 Feb 26;6(2):e0005881. doi: 10.1371/journal.pgph.0005881 (PMC12944787; doi:10.1371/journal.pgph.0005881)
Supplement: Attachment — Submitted filename: Response to review.docx [file pgph.0005881.s005.docx]

**A point-by-point response to reviewers**

**Comments from Reviewer # 1**

The manuscript presents valuable insights into the trends and disparities in severe anthropometric failures among children under five in Bangladesh using nationally representative survey data. The strength of the study lies in its comprehensive assessment of the Composite Index of Severe Anthropometric Failure (CISAF) and its focus on socioeconomic inequalities. However, several areas require revision to enhance the manuscript's clarity and policy relevance.

**Response:** We thank the reviewers for their careful evaluation of our manuscript and their constructive suggestions. We have addressed all comments point by point, with revisions made in the manuscript as indicated.

**Comment 1:** Firstly, the introduction would benefit from a more compelling articulation of the global significance of childhood severe undernutrition and the rationale for adopting CISAF over traditional indicators. A clearer depiction of the study’s contribution to existing literature would strengthen the context. Cite the following studies:

https://doi.org/10.1016/j.nut.2021.111565

https://doi.org/10.1108/NFS-03-2023-0055

**Response:** We thank the reviewer for this helpful suggestion. We have revised the Introduction to foreground the global burden and public-health significance of severe undernutrition, justify the use of CISAF over single indicators, and clarify our study’s contribution. Specifically, we now cite a recent systematic review that summarises key determinants and provides up-to-date global figures (https://doi.org/10.1016/j.nut.2021.111565). Due to an open-access barrier, we were unable to explore the full text of the second recommended paper (https://doi.org/10.1108/NFS-03-2023-0055). Updates were made in lines 63-68, 76-83, and 90-94.

**Comment 2:** Secondly, the methodology section presently lacks detail regarding sample selection, variable definitions, and the statistical procedures employed. A more explicit description of the analytical methods, including how inequalities were quantified, is essential for reproducibility.

**Response:** We appreciate this important observation. The Methods section has been revised to include more detailed information. Specifically, we now describe the two-stage stratified sampling design used in BDHS and MICS, the application of sampling weights, and the eligibility criteria for inclusion of children under five. Figure 1 describes the process of sample selection.

Variable definitions have been expanded, with explicit descriptions of CISAF construction, socioeconomic stratifiers, and covariates. The analytical approach is now presented step by step, covering descriptive statistics, logistic regression models, calculation of slope index of inequality (SII) and relative index of inequality (RII), and use of the Approximate Test of Homogeneity of odds ratios across survey years. These additions ensure methodological transparency and reproducibility (Lines 35; 98-119, 122, 130-132, 135-145, 151, 155-163, 180, 182, 187, 192-225, 282, 283, 287, 293, 302).

**Comment 3:** Furthermore, the paper should better extract and emphasize policy implications, such as targeted interventions for vulnerable groups identified by the study. A discussion of the limitations, including potential biases or unmeasured confounders, should be acknowledged to provide balanced conclusions.

**Response:** We agree with this important comment. The discussion has been revised to better highlight the policy implications of our findings, particularly the need for equity-focused interventions targeting socioeconomically disadvantaged households, children of less educated mothers, and those in rural areas. We now emphasise how CISAF can be integrated into nutrition surveillance systems to support more effective programme design. In addition, a limitations subsection has been updated. This acknowledges the cross-sectional nature of the surveys, potential reporting biases in morbidity data, missing anthropometry in BDHS 2022, and the inability to adjust for some unmeasured confounders (e.g., dietary diversity, household food security) (lines: 43-48, 384-389, 393-406).

**Comment 4:** Overall, addressing these points will significantly improve the manuscript's clarity, policy relevance, and scholarly rigor.

**Response:** We thank the reviewer for this encouraging summary comment. We have carefully revised the manuscript in line with all suggestions, including strengthening the Introduction with a clearer global context and rationale for CISAF, expanding the Methods with detailed descriptions of sampling, variables, and analytical procedures, and refining the Discussion to highlight both policy implications and study limitations.
