# Supplementary material for: National trends and socioeconomic inequalities in the Composite Index of Severe Anthropometric Failure among children under five in Bangladesh
Source: PLOS Glob Public Health. 2026 Feb 26;6(2):e0005881. doi: 10.1371/journal.pgph.0005881 (PMC12944787; doi:10.1371/journal.pgph.0005881)
Supplement: Attachment — Submitted filename: Response to review_r02.docx [file pgph.0005881.s006.docx]

**A point-by-point response to reviewers**

**Reviewer #1:**

**Comment 1:** The authors have improved the manuscript by clarifying data presentation and strengthening methodological details. The analysis of trends and inequalities in severe anthropometric failure using national survey data offers valuable insights into child nutrition in Bangladesh. However, key issues remain. The manuscript lacks a clear discussion of policy implications and practical applications of the findings. Explicitly linking results to national nutrition policies or targeted interventions would enhance its relevance. Overall, while the manuscript has advanced, a more comprehensive and policy-oriented discussion is needed to increase its impact and usefulness for stakeholders addressing child undernutrition in Bangladesh.

**Response:** Thank you for this constructive observation. We agree that a clearer articulation of policy implications would improve the manuscript’s relevance for decision-makers. We have now added a dedicated paragraph in the Discussion that explicitly links our findings to ongoing national nutrition strategies, including the National Nutrition Services Operational Plan and the National Strategy on Prevention of Stunting (lines 404-413).

**Reviewer #2:**

**Comment 1:** The comments have adequately addressed and the manuscript is ready for publication.

**Response:** We appreciate the positive assessment. We are grateful for the careful review and for the guidance that strengthened the manuscript.

**Reviewer #3:**

Thank you very much for giving me the opportunity to review the manuscript “National Trends and Inequalities in Severe Anthropometric Failures in Bangladesh: Evidence from National Surveys”. The relevance of this paper is notable, particularly in the context of Bangladesh. It utilises nationally representative data to explore nutritional disparities between boys and girls under the age of five. This research can increase scientific understanding. However, I recommend a thorough major revision to improve the manuscript’s presentation and overall impact.

**Response:** We thank the reviewer for the careful reading of the manuscript and for the recognition of its relevance in the context of Bangladesh. We appreciate the acknowledgment of the study’s contribution through the use of nationally representative data. We have addressed all points raised in the review and have revised the manuscript to improve clarity, organisation, and scientific impact (lines 1-2).

REVIEWER COMMENTS

SECTION-WISE OBSERVATION

**Comment 1:** Title: This is partially acceptable

• It lacks relevance and does not adequately reflect the study's objective. I recommend revising the title to better align with the content of the research. Additionally, while the study focuses on CISAF, the mention of "Severe Anthropometric Failures" seems misplaced and should be clarified or adjusted accordingly.

**Response:** We thank the reviewer for this important observation. We agree that the title should reflect the study aim more clearly. The original wording may imply a broader anthropometric focus, although the analysis centres on CISAF. We have therefore revised the title to present the outcome measure directly and to improve alignment with the objectives (lines 1-2).

**Comment 2:** Keywords: This is partially acceptable

• Please avoid repeating terms from the title in the keywords section.

• Acronyms such as "DHS," "MICS," "SII," and "RII" should be spelled out to enhance understanding, as they may not be familiar to all readers.

**Response:** We thank the reviewer for this helpful guidance. We agree that repeating terms from the title offers limited value and that spelling out acronyms will improve clarity for a wider readership. We have revised the keyword list by removing repeated terms and replacing acronyms with their full forms (lines 53-54).

**Comment 3:** Abstract: Partially Acceptable; moderate corrections are required

• You have collected data from BDHS 2017-18, MICS 2019, and BDHS 2022, leading to a trend analysis covering a relatively short time span of only five years. For a more robust analysis, I recommend using data spanning at least a decade.

**Response:** We thank the reviewer for this useful suggestion. We agree that a longer window can offer a broader historical view. The present study focuses on the three most recent nationally representative surveys because the objective was to assess the current situation and recent disparities in severe anthropometric failure. These surveys also reflect the most up-to-date programme period in Bangladesh, which makes the findings more relevant for ongoing policy planning. Extending the analysis to earlier years would shift the study beyond its intended scope and would require additional analytical work that is not feasible at this stage. We have also added this as a limitation (lines 32-33, 335-336, 398-399).

**Comment 4:** • Is this study intended as a longitudinal study? You note that “Higher odds of CISAF were associated with increasing age of the child (aOR: 1.03, 1.10 and 1.02, respectively).” suggesting that children become CISAF as they grow older. This warrants further clarification or write the sentence correctly.

**Response:** We thank the reviewer for raising this important point. The study is not longitudinal. All findings are based on cross-sectional survey data. The association with age reflects differences between age groups at the time of the survey rather than progression within individuals. We have revised the sentence to remove any implication of temporal change and to clarify that the results describe cross-sectional patterns only. Additional changes were made to avoid causal interpretation (lines 40-42, 358-359, 370-371).

**Comment 5:** • Regarding your statement, “However, lower odds were linked to wealthier families and higher education of the mother.” while maternal education is undoubtedly significant, it would enhance your analysis to include paternal education as well.

**Response:** We thank the reviewer for this valuable suggestion. We agree that paternal education is an important socioeconomic factor. However, paternal education was not included because this variable is not consistently available for all under-five children across the three survey rounds used in this study (not directly available in the child dataset of MICS 2019). To maintain comparability between BDHS 2017-18, MICS 2019, and BDHS 2022, the analysis was restricted to variables that were reported for all eligible children in a harmonised format.

**Comment 6:** • I suggest clarifying and refining the conclusion for a more precise presentation of your findings.

**Response:** We appreciate the reviewer’s suggestion. We have revised the conclusion to present the findings more clearly and concisely. The updated version highlights the key results, addresses socioeconomic disparities, and summarises the public health relevance without implying causality. The revised conclusion offers a more precise summary that aligns with the study objectives (lines 46-52).

**Comment 7:** Introduction: Acceptable, but major corrections are required

• In the sentence "To overcome this, Svedberg proposed the Composite Index of Anthropometric Failure (CIAF), which incorporates all forms of failure.[1]" the reference is cited as Anik et al. (2021). It seems more appropriate for this claim to be attributed to Svedberg. Could you please clarify this?

**Response:** We thank the reviewer for this helpful observation. Anik et al. provided a secondary explanation of CIAF, but the original development of the index was introduced by Svedberg. We have therefore added the primary reference to ensure accurate attribution. The revised sentence now cites Svedberg’s original work (lines 81-82).

**Comment 8:** • In your discussion regarding CISAF, you mention "Later, Vollmer et al. (2017) introduced the...in resource-poor settings.[5]". However, the reference here is listed as Saif and Anwar (2023). Please review this and ensure that proper attribution is given.

**Response:** We thank the reviewer for identifying this inconsistency. The sentence should indeed attribute the work to Vollmer et al. We have corrected the citation to reflect the original source and updated the reference list accordingly (lines 85).

**Comment 9:** • I recommend that you recheck all the references thoroughly, as the arrangement appears to be inconsistent. A systematic review of citations would enhance the manuscript’s credibility.

**Response:** Thank you for the comment. We have rechecked all the references and made several changes. We have added a few references, removed duplicate ones and cross-checked them.

**Comment 10:** • It may be beneficial to include further references to support your study. This could help substantiate your claims and provide a broader context for your findings.

Response: Thank you for the comment. We have included further references to support the study’s findings.

**Comment 11:** Objective: Partially Acceptable; minor corrections are required

• The objective should be revised to provide a clear statement that reflects the title of the study.

**Response**: We thank the reviewer for this helpful suggestion. We agree that the objective should closely match the title and scope of the study. We have revised the objective to provide a clear and direct statement that reflects the focus on national trends and socioeconomic inequalities in CISAF (lines 96-100, 328) .

**Comment 12:** • The text states, "Using three recent nationally representative surveys..." However, there is no mention of the names of these surveys. It would be helpful to include this information to provide clarity and context for the reader.

**Response:** Thank you for the comment. We have now mentioned the surveys (the Bangladesh Demographic and Health Survey (BDHS) 2017-18, the Multiple Indicator Cluster Survey (MICS) 2019, and the BDHS 2022) to provide clarity and context (line 96-100).

**Comment 13:** Methods: Partially Acceptable; moderate corrections are required

• The definition of implausible values for Z-scores appears to deviate from standard ranges, instead of "Implausible values were defined as weight-for-height Z-score (WHZ) <-5 or >5 SD, height-for-age Z-score (HAZ) <-5 or >5 SD, and weight-for-age Z-score (WAZ) <-6 or >5 SD.". Please refer to the WHO Multicentre Growth Reference Study Group's guidelines: "WHO child growth standards: length/height-for-age, weight-for-age, weight-for-length and body mass index for age: methods and development. Geneva: World Health Organization, 2006." Specifically, implausible values should be based on accepted thresholds for weight-for-height Z-score (WHZ), height-for-age Z-score (HAZ), and weight-for-age Z-score (WAZ).

**Response:** We thank the reviewer for this helpful clarification. In our analysis, we did not manually apply Z-score thresholds. Instead, we used the survey-generated WHO flag variables included in each DHS and MICS dataset. These flags follow the WHO 2006 Growth Standards and identify biologically implausible anthropometric values based on the accepted Z-score cut-offs. We have revised the Methods text to explain this more clearly. We sincerely apologise for the oversight and thank the reviewer for pointing it out (line 130-135).

**Comment 14:** • I noticed inconsistencies in the terminology used to refer to the target population. It would be beneficial to adopt a single term throughout the manuscript, such as "under-five children," "U5C," or "under five," to enhance clarity.

**Response:** We thank the reviewer for this observation. We reviewed the full manuscript and confirmed that the term “children under five” is used consistently throughout the text. We have retained this wording across all sections to ensure clarity and uniformity. No additional terminology now appears in the manuscript (lines 2, 29, 46, 100, 114, 137, 142, 146, 242, 244, 246, 329, 416) .

**Comment 15:** • The focus of this study is on "Inequalities"; however, the sex of the child (male/female) is utilized as an independent variable. I suggest a more thorough exploration of the potential implications of this variable in the context of inequalities.

**Response:** We thank the reviewer for this helpful suggestion. The principal aim of our study is to quantify socioeconomic inequalities in CISAF. Child sex was included as a covariate to adjust for potential differences in nutritional risk between boys and girls and to avoid confounding when estimating wealth- and education-related inequalities. We did not treat sex as a primary axis of inequality in our SII and RII analyses. We have clarified this choice in the Methods and added a brief interpretation in the Discussion (line 225-228, 400-410).

**Comment 16:** • Additionally, I recommend including a section labelled "Study variables" that distinctly outlines the "Outcome variable" and the "Independent variable." It would also be beneficial to provide "Operational definitions for the variables" separately to enhance understanding.

**Response:** We thank the reviewer for this constructive suggestion. We agree that presenting a clear “Study variables” section improves readability and strengthens the structure of the Methods. We have therefore added a new header titled *Study variable*, under which the outcome and independent variables are described. We also created a short subsection providing operational definitions to enhance clarity (line 139, 152, 161, 400-401).

**Comment 17:** Results: Partially Acceptable, major corrections are required

• I recommend conducting the analysis separately for boys and girls, as the focus is on "inequalities." This approach may reveal factors that are specifically related to each gender, providing a deeper understanding of the issues at hand.

**Response**: We thank the reviewer for this thoughtful suggestion. We agree that sex-stratified analyses can offer important insights. The present study focuses on socioeconomic inequalities in CISAF and applies SII and RII as the primary inequality measures. Conducting sex-stratified analyses would require additional modelling and recalculation of inequality indices, which fall outside the scope of the current study.

**Comment 18:** Discussion: Acceptable; minor corrections are required

• It may be beneficial to include further references to support your study.

Response: Thank you for the valuable comment. We have added further references in the discussion section.

**Comment 19:** Conclusion: This is acceptable

**Response:** We thank the reviewer for this positive assessment. We appreciate the careful review and are pleased that the revised conclusion meets the expectations.

**Comment 20:** References: This is acceptable

**Response:** We thank the reviewer for the comment.

**Comment 21:** OVERALL OBSERVATION

The suggestion for improving the language to avoid the use of "we" or "our" in the manuscript is well-founded. By opting for a more formal and objective tone, the authors can enhance the clarity and professionalism of their work.

**Response:** Thank you for the suggestion. We have made suggested changes throughout the manuscript (lines 1-2; 28-30; 103-104; 137-138, 142, 195-196, 242, 256, 332, 340, 350, 351-353, 370, 403).
